# Use of a special bioreactor for the cultivation of a new flexible polyurethane scaffold for aortic valve tissue engineering

**DOI:** 10.1186/1475-925X-11-92

**Published:** 2012-12-04

**Authors:** Genoveva Aleksieva, Trixi Hollweck, Nikolaus Thierfelder, Ulrike Haas, Fabian Koenig, Cornelia Fano, Martin Dauner, Erich Wintermantel, Bruno Reichart, Christoph Schmitz, Bassil Akra

**Affiliations:** 1 Department of Cardiac Surgery, Medical Center Munich University, Marchioninistraße 15, Munich, 81377, Germany; 2 Institute of Textile Technology and Process Engineering, Körschtalstraße 26, Denkendorf, 73770, Germany; 3 Chair of Medical Engineering, Technical University of Munich, Boltzmannstrasse 15, Garching, 85748, Germany

**Keywords:** Tissue engineering, Heart valve, Polyurethane scaffold, Static cultivation, Dynamic cultivation

## Abstract

**Background:**

Tissue engineering represents a promising new method for treating heart valve diseases. The aim of this study was evaluate the importance of conditioning procedures of tissue engineered polyurethane heart valve prostheses by the comparison of static and dynamic cultivation methods.

**Methods:**

Human vascular endothelial cells (ECs) and fibroblasts (FBs) were obtained from saphenous vein segments. Polyurethane scaffolds (n = 10) were primarily seeded with FBs and subsequently with ECs, followed by different cultivation methods of cell layers (A: static, B: dynamic). Group A was statically cultivated for 6 days. Group B was exposed to low flow conditions (t_1_= 3 days at 750 ml/min, t_2_= 2 days at 1100 ml/min) in a newly developed conditioning bioreactor. Samples were taken after static and dynamic cultivation and were analyzed by scanning electron microscopy (SEM), immunohistochemistry (IHC), and real time polymerase chain reaction (RT-PCR).

**Results:**

SEM results showed a high density of adherent cells on the surface valves from both groups. However, better cell distribution and cell behavior was detected in Group B. IHC staining against CD31 and TE-7 revealed a positive reaction in both groups. Higher expression of extracellular matrix (ICAM, Collagen IV) was observed in Group B. RT- PCR demonstrated a higher expression of inflammatory Cytokines in Group B.

**Conclusion:**

While conventional cultivation method can be used for the development of tissue engineered heart valves. Better results can be obtained by performing a conditioning step that may improve the tolerance of cells to shear stress. The novel pulsatile bioreactor offers an adequate tool for in vitro improvement of mechanical properties of tissue engineered cardiovascular prostheses.

## Background

Valve replacement represents the most common surgical therapy for end staged valvular diseases with an estimated number of 275.000 procedures performed annually worldwide [1]. The commonly used artificial heart valves are mechanical or biological prostheses. According to the American Heart Association, mechanical heart valves are recommended for patient under 60 years of age [2]. However, the increased risk of postoperative hemorrhage, thromboembolism, and drug-drug interactions affect patients’ quality of life [2]. Biological valves are accompanied by a low risk for thromboembolism and endocarditis, and offer growth potential for pediatric patients [3,4]. However, biological valves are associated with different major complications such as deterioration of valve structure, graft calcification, limited durability, and affinity to immunological response [2]. Tissue engineered heart valves (TEHVs) are a promising approach to overcome the limitations of conventional heart valve prostheses. Tissue engineering generally aims for the *in-vitro* creation of viable neo-tissue indistinguishable from native tissue [5]. Biological and engineering challenges are focused on three principal components that comprise the “cell–scaffold–bioreactor system” [6]. An adequate combination of these components could be the ideal solution for heart valve grafting leading to biocompatibility, non-thrombogenicity, non-teratogenicity, long-term durability and growth potential of TEHVs [7]. The aim of this study was to compare static cultivation (SC) and dynamic cultivation (DC) of endothelial cells (ECs) and fibroblasts (FBs) seeded onto polyurethane heart valve scaffolds by evaluating cell confluency, extracellular matrix (ECM) formation and inflammatory response.

## Methods

### Cell isolation

Cells were isolated from human saphenous vein segments left over from cardiac surgery interventions. Tissue samples were only taken with the patients’ informed consent and were further used in an anonymous fashion with no individual-related data. Veins were cannulated and rinsed with aliquots of 500 ml M199 (Biochrom AG, Berlin, Germany) supplemented with 1 ml Heparin (5000 i.E.; Ratiopharm GmbH, Ulm, Germany) and 5 ml Gentamycin (10 mg/ml; Invitrogen AG, Darmstadt, Germany). For EC isolation, segments were incubated with trypsin/EDTA-solution (10x; Sigma-Aldrich GmbH, Taufkirchen, Germany) for 25 min at 37°C / 5% CO_2._ For FBs isolation, veins were subsequently flushed with 2 mg/ml collagenase type II (Worthington Biochemical Corporation / CellSystems GmbH, St. Katharinen, Germany) in human serum albumin (200 g/l; Baxter GmbH, Unterschleißheim, Germany) and incubated for 30 min. Cell suspensions were centrifuged at 750 rpm for 10 min, and cultured in endothelial cell growth medium (Promocell GmbH, Heidelberg, Germany) supplemented with 6% FCS (Lonza GmbH, Köln, Germany) and 0.2% Penicillin/Streptomycin (Sigma Aldrich GmbH, Hamburg, Germany) and fibroblast growth medium (Promocell GmbH, Heidelberg, Germany) supplemented with 11% FCS and 0.2% Penicillin/Streptomycin, respectively. Medium was changed every second day. Cells were passaged at confluency.

### Phenotypic characterization of isolated cells

Morphological and immunocytological analysis were performed to characterize isolated cell types. ECs were identified by their typical cobblestone morphology. FBs were identified by a characteristic elongated spindle-shaped appearance with several extensions. For immunocytological verification of ECs and FBs, 35.000 cells/cm^2^ were cultured in an 8-well culture slide (BD Bioscience, Bedford, USA) until confluency. Vascular cells were stained against EC-specific CD31 (0.14 μg/ml; Dianova GmbH, Hamburg, Germany) and FB-specific TE-7 (0.67 μg/ml, Millipore Corporation BioScience Division, Temecula, CA, USA), respectively according to manufacturer’s protocol using EnVision^™^ + Dual Link System-HRP (Dako Deutschland GmbH, Hamburg, Germany). Briefly, cells were fixed at – 80°C in 96% ethanol. The staining procedure was performed at room temperature (RT). After rinsing with phosphate buffered saline (PBS; Biochrom AG, Berlin, Germany) and blocking for endogenous peroxidase using 30% H_2_O_2,_ cells were incubated with the primary antibody for 30 min. The procedure was completed by incubation with EnVision^™^ + Dual Link System- HRP (Dako Deutschland GmbH, Hamburg, Germany) for 30 min; and AEC- Peroxidase-Substrate (Vector Laboratories, Inc., Burlingame, CA, USA) incubation for 10 min. Counterstaining was performed using 25% Mayer’s Haemalaun (Merck KGaA, Darmstadt, Germany) in PBS for 3 min at RT. Controls for non-specific binding of biotinylated link were performed by excluding the primary antibody. The stained cells were analyzed using bright field microscopy (Leica DMR microscope, Leica Microsystems GmbH, Wetzlar, Germany).

### Fabrication of polyurethane heart valve prosthesis (PHVP)

PHVPs (h = 55 mm, d = 18 mm) were manufactured by ITV-Denkendorf (Denkendorf, Germany) using a polyurethane spraying technique (patent DE 28 06 030 C2). Randomly oriented PU fibres formed a sheet with a thickness of 0.3 mm. For seeding purpose, PHVP was γ-sterilized at 10 kGy according to a certified sterilization procedure.

### Seeding procedure

**Figure 1:**
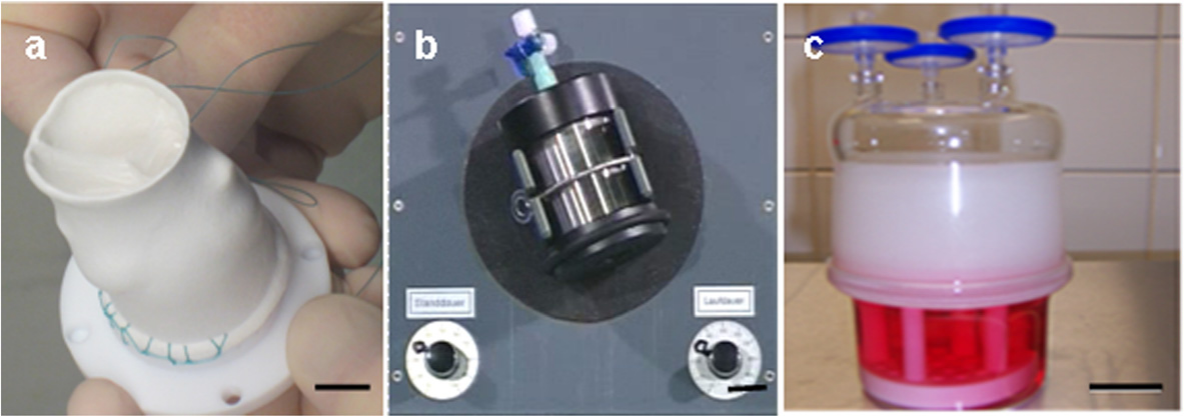
**Seeding of PHVPs.** PHVPs were sutured to a Teflon® fixation unit (**a**) and were consecutively seeded with FBs and ECs using a 3D-rotating bioreactor (**b**) for 24 h at 37°C / 5% CO_2_. After the dynamic seeding procedure, PHVPs were statically cultivated for 6 d (SC group) and 1 d (DC group), respectively at 37°C / 5% CO_2_ in a glass container (**c**). Scale bars: a = 10 mm, b = 20 mm, c = 40 mm.

PHVPs were sutured to a Teflon® fixation unit (Figure 1a, manufactured in-house) and were seeded as previously described [8]. Briefly, PHVPs were initially seeded with FBs (1.5 × 10^6^ cells/cm^2^) using a 3D- rotating seeding device (Figure 1b; manufactured in-house) for 24 h (running phase: 2.5 min; holding phase: 30 min), at 37°C / 5% CO_2_ followed by a stationary cultivation phase of 6 d (SC group) and 1 d (DC group), respectively at 37°C / 5% CO_2_ in a glass container (Figure 1c). Cell medium was changed every two days. Colonization of ECs was analogously performed. 

### Cultivation procedures

For *SC,* seeded PHVPs (n = 5) were cultured for 6 d at 37°C / 5% CO_2_ in a glass container. This procedure is analog the stationary cultivation phase of the seeding procedure shown in Figure 1c.

**Figure 2:**
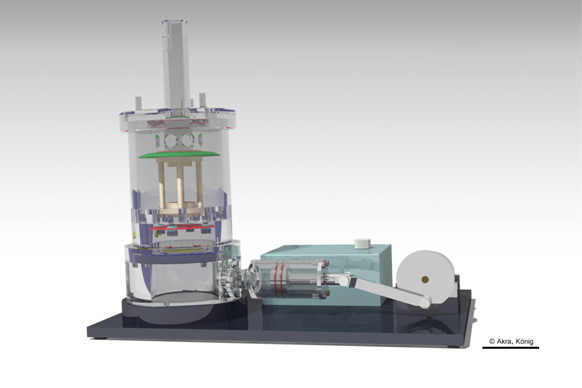
**DC of seeded PHVPs.** Seeded PHVPs in the group DC were conditioned in a pulsatile bioreactor with an endoscopic monitoring unit [9] for 3 d at 750 ml/min and 2 d at 1100 ml/min. Scale bar = 50 mm.

For *DC*, PHVPs (n = 5) were conditioned in a novel pulsatile bioreactor with an endoscopic monitoring unit (Figure 2; EU-Patent pending EP10166094; manufactured in-house, [9]) for 3 d at 750 ml/min and 2 d at 1100 ml/min medium flow, after FB seeding and after EC seeding. For the conditioning of FB seeded PHVP, fibroblast growth medium supplemented with 11% FCS and 0.2% Penicillin/Streptomycin were used. FB+EC seeded PHVP were conditioned using endothelial cell growth supplemented with 6% FCS and 0.2% Penicillin/Streptomycin. The viscosity of the media was 0.738 mPas ± 0.078 mPas. Cell medium was partially changed every two days. For further analysis, samples were taken from native as well as from seeded PHVPs after SC and after DC. Samples were taken from the supravalvular, valvular and subvalvular region of the aortic wall as well as from the valvular leaflets. 

### Immunohistochemistry (IHC)

Immunohistochemical stainings were performed to differentiate between FB and EC layers on seeded PHVPs (n = 10). Samples were fixed in 4% formalin (Microcos GmbH, Garching, Germany) for 10 d, embedded in paraffin and sectioned at 10 μm. Specimen were deparaffinized in Xylene (Carl Roth GmbH + Co. KG, Karlsruhe, Germany), rehydrated by an descending ethanol (Merck KGaA, Darmstadt, Germany) series and permeabilized with 0.5% Triton-X (Sigma Aldrich Chemie GmbH, Taufkirchen, Germany) in PBS for 10 min at RT. Samples for staining against VE-Cadherin, Connexin-43, Fibronectin and Collagen IV were exposed to 10% Protease (Dako Deutschland GmbH, Hamburg, Germany) in distilled water (Ampuwa, Fresenius Kabi Deutschland GmbH, Bad Homburg v.d. H., Germany) for 10 min at RT. For proteolysis of Fibronectin, Collagen IV, and SMC-Myosin, specimens were boiled in 0.1 mM EDTA buffer (pH = 8.0, Sigma Aldrich Chemie GmbH, Taufkirchen, Germany) or in Target Retrieval solution (pH = 6.0, Dako Deutschland GmbH, Hamburg, Germany) for 15 min. For demasking of α- Actin, samples were boiled in 10 mM Tris/1 mM EDTA solution (pH = 9.0, Sigma Aldrich Chemie GmbH, Taufkirchen, Germany) for 15 min. After blocking for endogenous peroxidase using 0.4% H_2_O_2_ in PBS, samples were incubated overnight at 4°C with primary antibodies against VCAM (200 μg/ml), ICAM, SMC-Myosin (0.954 mg/ml; Dako Deutschland GmbH, Hamburg, Germany), Fibronectin (0.6 mg/ml; Sigma Aldrich Chemie GmbH, Taufkirchen, Germany), TE-7 (0.1 mg/ml; Millipore GmbH, Schwalbach / Ts.,Germany), Connexin- 43 (1 μg/ml; Millipore GmbH, Schwalbach/Ts., Germany), VE- Cadherin (0.2 mg/ml; Beckmann Coulter Inc., Marseille, France), Collagen IV (5.4 mg/ml; Sigma Aldrich Chemie GmbH, Taufkirchen, Germany), CD31 (0.2 mg/ml; Dako Deutschland GmbH, Hamburg, Germany) and α- Actin (44 μg/ml; Dako Deutschland GmbH, Hamburg, Germany). Specimens were incubated with EnVision™ + Dual Link System-HRP (Dako Deutschland GmbH, Hamburg, Germany), for 10 min, followed by AEC labelling using AEC-Peroxidase-Substrate Kit (Vector Laboratories, Inc., Burlingame, CA, USA) for 10 min at RT. Counterstaining was performed for 3 min at RT using Mayer’s Hemalaun (Merck KgaA, Darmstadt, Germany). Controls for non-specific binding of biotinylated link were performed by excluding primary antibodies. Sections in duplicates of each region were qualitative observed using bright field microscopy (Leica DMR microscope, Leica Microsystems GmbH, Wetzlar, Germany) in four fields of vision. The intensity of IHC staining was analyzed by a minimum of two experts without being blinded to intervention and were classified as high (+++), medium (++), low (+) and absent (0).

### Scanning electron microscopy (SEM)

Samples were fixed in 456 ml aqua bidest supplemented with 0.75 ml 1 N hydrochloric acid (Titrisol, Merck KGaA, Darmstadt, Germany), 43.5 ml glutaraldehyd (Sigma-Aldrich Chemie GmbH, Steinheim, Germany) and 5.65 g sodium cocodylate trihydrate (Sigma-Aldrich Chemie GmbH, Steinheim, Germany) at 4°C for 48 h. Dehydration of fixed specimens was performed by an ascending ethanol series (30%, 50%, 70%, 96%) and after which the samples were place in 100% acetone (Merck KGaA, Darmstadt, Germany). After sample drying at the critical point, specimens were coated with gold for 180 s at 10^-5^ and examined under a scanning electron microscope (Carl Zeiss MikroImaging GmbH, Göttingen, Germany). Unseeded PHVP specimen served as negative controls.

### Real time PCR (RT- PCR)

For detection of cytokine expression after SC and DC, RT-PCR was performed according to manufacturer’s protocols. Briefly, RNA was isolated from samples stored in liquid nitrogen using RNAeasy Plus Mini Kit (Qiagen, GmbH, Hilden, Germany). RNA purity and quantity was photometrically (BioPhotometer, Eppendorf AG, Hamburg, Germany) assessed. QuantiTect Reverse Transcription Kit (Qiagen, GmbH, Hilden, Germany) was applied for reverse transcription. Rotor-Gene Q 2plex System (35 cycles; Qiagen GmbH, Hilden, Germany) and QuantiFast SYBR Green PCR Kit (Qiagen GmbH, Hilden, Germany) were used to determine IL-1a, IL-6, IL-8, MCP-1, VCAM and GAPDH (QuantiTect Primer Assay, Qiagen GmbH, Hilden, Germany) expression. A standard curve was generated to determine the primer-dilution. Negative controls without sample material were included for all PCR measurements. Resulting Ct-values were normalized to the housekeeping gene GAPDH. PCR-product specificity was verified by melt curve analysis and gel electrophoresis (FlashGel System, Lonza GmbH, Basel, Switzerland).

### Statistical analysis

All values are expressed as mean ± standard deviation. Student’s t-test was performed for comparison of data of unpaired samples. All tests are one-tailed; the probability value p < 0.05 was considered significant.

## Results

### Cell confluence

**Figure 3:**
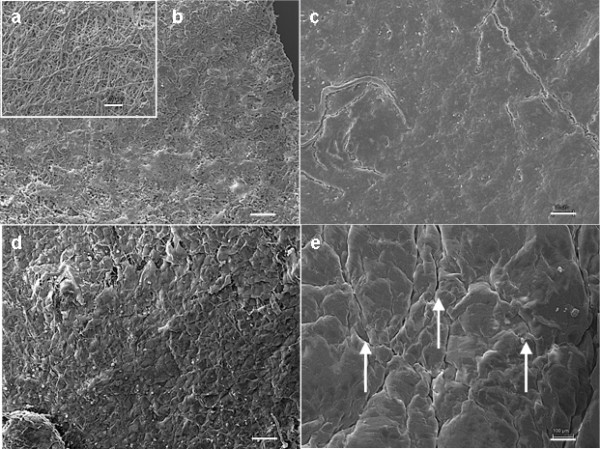
**SEM analysis of PUHVs in different processing states.** Native PHVPs demonstrate disordered fibers (**a**). FB seeded PHVPs revealed a rough confluent cellular coverage after SC (**b**) and a smoothed cellular surface after DC (**c**). FB and EC seeded PUHVs reveal a confluent cell layer with an EC-typical cobblestone morphology after SC (**d**). DC results in cell alignment following flow direction (**e**). These are representatives of ten independent experiments. Scale bars = 100 μm.

Seeding and cultivation of PHVPs were performed as described in materials and methods section. SEM analysis (Figure 3) of native samples (a) showed randomly orientated fibres. FB seeded PHVPs revealed a rough confluent cellular coverage after SC (b) and a smoothed cellular surface after DC (c). FB and EC seeded PHVPs showed a confluent cell layer after SC (d) and DC (e). In addition, the typical cobblestone morphology indicates an endothelial layer after SC and DC. Moreover, flow conditions (t_1_= 3 d at 750 ml/min, t_2_= 2 d at 1100 ml/min) influence cell alignment; ECs of the internal side of the PHVPs were orientated in flow direction (e).

### Protein expression

**Figure 4:**
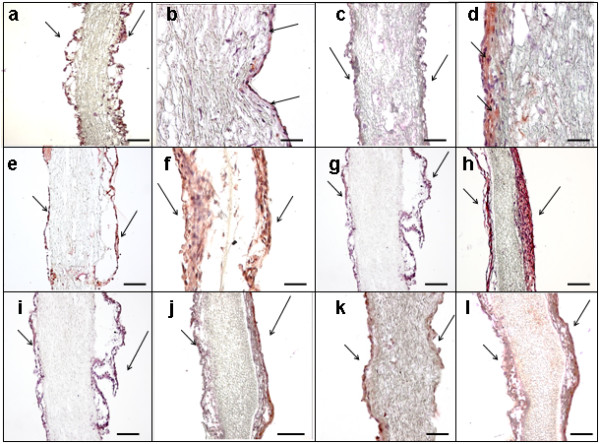
**IHC analysis of PUHVs in different processing states.** Seeded PUHVs reveal an EC presence (brown; arrows) after SC (**a**) and DC (**b**) SC (**c**) and DC (**d**) also result in the formation of fibroblast multilayer (brown; arrows). A lower expression of ICAM was detected after SC (e; brown, arrows) than after DC (f; brown, arrows). VCAM was also expressed less after SC (g; brown, arrows) than after DC (h; brown, arrows). A lower expression of Collagen IV was observed after SC (i; brown, arrows) compared to DC (j; brown, arrows); VE- Cadherin was also expressed less after SC (k; brown, arrows) than after DC (l; brown, arrows). Cell nuclei were stained with haemalaun (purple). These are representatives of ten independent experiments. Scale bars: a, c, e-l = 150 μm, b, d = 50 μm.

IHC examination was performed to compare cellular coverage and ECM formation after SC and DC. Cell nuclei were stained with haemalaun (purple). As shown in Figure 4, staining against CD31 (brown; arrows) revealed a positive reaction at both culture conditions (a: SC, b: DC), indicating EC presence. Fibroblasts were detected after SC (c) and DC (d) in a continuous multilayer by staining against TE-7 (brown, arrows). Comparison of cellular adhesion molecules demonstrated a lower expression of ICAM after SC (e; brown, arrows) than after DC (f; brown, arrows). VCAM was also expressed less after SC (g; brown, arrows) than after DC (h; brown, arrows). A lower expression of Collagen IV was observed after SC (i; brown, arrows) compared to DC (j; brown, arrows); VE- Cadherin was also expressed less after SC (k; brown, arrows) than after DC (l; brown, arrows). Controls for non-specific chromogen binding displayed negligible staining for antigens (data not shown).

### Gene expression

**Figure 5:**
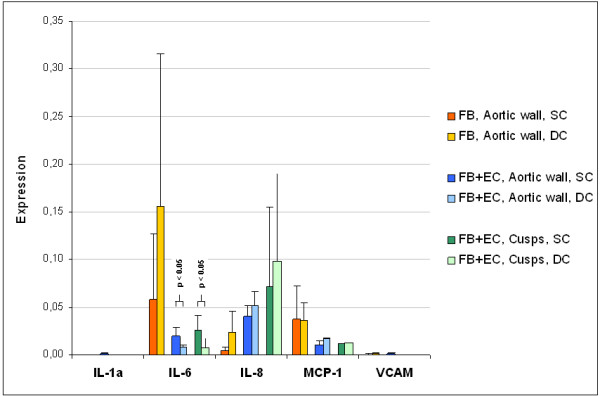
**Overview of the PCR results.** The bar chart shows the expression of several cytokines / chemokines in various stages of valve colonization. Gene expression was normalized to the expression of the housekeeping gene GAPDH. Results are given as the mean values of three independent experiments ± standard deviation.

Figure 5 illustrates the mean values of gene expression in the aortic wall and cusps after SC and DC. Gene expression was normalized to the expression of the housekeeping gene GAPDH. The analysis of the aortic wall segments and the cusps of the heart valves proportionately showed equivalent gene expressions after SC and DC for all cytokines. The analysis of IL-1a and VCAM revealed a negligible expression after SC and DC. In both cultivation procedures seeding of EC results in a decrease of IL-6 (SC: -66%, DC: -95%) and MCP-1 (SC: -71%, DC: -56%) expression while IL-8 (SC: + 868%, DC: + 123%) was expressed to a higher level. The comparison of the SC and DC of FB+EC seeded aortic wall showed a lower expression of IL-6 (− 59%) and an increase of IL-8 (+ 29%) and MCP-1 (+ 51%) expression after DC. FB+EC seeded cusps showed a lower expression of IL-6 (− 72%), an increase of IL-8 (+ 37%) expression and a comparable MCP-1 expression (+ 10%) after DC compared to SC.

## Discussion

Tissue engineering is an emerging field focused on the development of bioartificial substitues to restore, maintain, or improve tissue function. These applications are the key for the future treatment of many diseases [10]. Currently, in tissue engineering are several scaffolds materials under investigation. Synthetic, non-degradable polymers like polyurethane are mainly characterized by their structural resistance, a three dimensional form with defined pore sizes, non-immunogenic and anti-thrombotic properties [11]. Within the monomeric unit, moieties could be substituted by different groups, resulting in versatile properties. Fabrication of hydrolytic stable PU already led to the development of different implants like vascular grafts, artificial heart valves and catheters [11,12]. Cells needed for the development of the tissue engineered heart valves can be obtained from a saphenous vein - vascular cells, be taken by a bone marrow biopsy - bone marrow stromal cells, from newborn patients, umbilical cord-derived cells or blood derived endothelial progenitor cells and chorionic villi-derived cells [13]. In our study, we obtained FBs and ECs from saphenous vein segments which were no longer required in coronary bypass operations. According to Schmidt et al., neither the proliferation in a monolayer nor the three-dimensional growth as tissue engineered constructs is influenced by the age of the cell donor [13], indicating that adult saphenous vein segments are an adequate cell source for tissue engineering applications. During the development of a cardiovascular tissue-engineered construct, a large challenge is the creation of a confluent and stable endothelial cell layer. Complications after the implantation of artificial grafts are caused in part by the lack of an intact endothelium [14]. The endothelial cell function has been described several times [15,16]. Consigny et al. showed a better adhesion and shear stress resistance of ECs on prosthetic vessels or heart valves pre-coated with different ECM proteins [16]. Although this coating improves cell adhesion, the integrity of the coating is compromised at high flow rates [17]. Another strategy to enhance the adhesion of ECs is the pre-seeding of prosthesis with vascular FBs, mimicking conditions *in vivo*[8]. In addition, the fibrillar structure of PHVPs is similar to collagen, the main component of native ECM, and may support cell adhesion. Moreover, *In vivo*, cellular phenotype, morphology, and proliferation are affected by mechanical, electrical and chemical signals [18]. If these signals are inappropriate or absent, cells lose their ability to develop an ECM and to form organized tissues [19]. Thus, the simulation of physiological conditions, such as shear stress, plays an important role in the development of tissue engineered constructs [20,21]. For the fabrication of vascular grafts, Syedain *et al.* and Tschoeke *et al.* demonstrated the expression of ECM components by human dermal fibroblast and ovine arterial myofibroblasts in fibrin gel under dynamic culture conditions generated by a pulsed flow-stretch bioreactor and a pulsatile bioreactor, respectively [22,23].

In heart valve fabrication, bioreactors for tissue formation under dynamic culture conditions have been reported several times [24,25]. Ramaswamy *et al.* described a large collagen mass production after the use of simulated pulmonary artery conditions using an organ-level heart valve bioreactor [26]. The stimulation of human dermal fibroblasts seeded onto a decellularized porcine matrix by a pneumatic flow bioreactor, resulting in the synthesis of ECM proteins was shown by Zeltinger *et al.*[27]. Mol et al. demonstrated that dynamically strained leaflets reveal a more homogenous and denser cellular coverage than leaflets exposed to pre-strain only [25]. This is in line with results, generated in our study: SC and DC results in a confluent cell layer. In this context, numerous studies have reported the behaviour of ECs to flow shear stress in-vitro. ECs are constantly subjected to hemodynamic forces, including shear stresses that induce various functional changes in vascular endothelium. Initially, it was found that exposure of ECs to elevated shear stresses in-vitro caused them to align in the direction of flow [28]. In our study, after DC ECs were also orientated into flow direction after DC, indicating the adaption to shear stress. The higher expression of cellular adhesion molecules after DC illustrates the intensified formation of cell connecting molecules, due to the pulsatile conditioning process. Moreover, a higher expression of Collagen IV, VE-Cadherin and Fibronectin was observed after DC indicating the formation of an ECM, essential for tissue and organ morphogenesis, maintenance, and reconstruction following injury in association with constructive tissue remodeling [29]. However, shear stress as a result of the DC provoked a higher cytokine expression compared to SC [30]. EC are able to sense changes in the shear stress or flow forces and respond, for instance, by expression of pro-inflammatory cytokines [31]. These cytokines and/or chemokines play key roles in mediating inflammatory reactions [32]. Gerszten et al. already concluded that cytokines are important modulators of monocyte-endothelial interactions under flow conditions [33]. McGill et al. demonstrated that consecutive seeding of heart valve scaffolds with FB and EC results in a less inflammatory response after DC than singly seeding with EC [34]. Chiu et al. reported that a coculture of vascular ECs with vascular smooth muscle cells induces the expression of ICAM-1, VCAM-1, and E-selectin genes in ECs in the static condition, whereas the application of shear stress to ECs inhibits these coculture-induced gene expressions [35]. Our analysis of IL-1a and VCAM expression revealed a negligible expression after SC and DC of FB and EC seeded scaffolds. These findings are also described by McHale et al. and Murui et al., indicate a lower risk of inflammatory response and arteriosclerosis [36,37]. While IL-1a and VCAM were expressed to a lower level after DC, the expression of IL-8 increased after DC. Several studies have shown a correlation between IL-8 expression, arteriosclerosis and coagulation which is thought to be caused by monocyte activation, adhesion and transmigration across the endothelial barrier [34,38]. However, not only shear stress, but rather cell isolation from biopsies provokes stress symptoms. For example, the process of human islet isolation triggers a cascade of stressful events in the islets of Langerhans involving the production of proinflammatory molecules. Two of the major pathways responsible for cellular responses to stress, already occurs in pancreatic cells during the isolation procedure. The production and release of IL-6, IL-8 and MCP-1, were observed days after the isolation procedure in isolated purified islets [39]. Therefore, the next step will be long-term conditioning of our TEHVs for a better adaption of cells to shear stress after isolation and cultivation procedure and consequently to reduce inflammatory response.

## Conclusions

In conclusion, we demonstrate that DC is more effective than SC in generating TEHVs. DC supports ECM formation and homogeneity of the cellular coverage. The novel pulsatile bioreactor provides a strong tool for dynamic pre-conditioning of TEHVs.

## Abbreviations

AEC: 3-amino-9-ethylcarbazole; CD31: Cluster of differentiation 31; DC: Dynamic cultivation; ECs: Endothelial cells; ECM: Extracellular matrix; FBs: Fibroblasts; FCS: Fetal calf serum; GAPDH: Glyceraldehyde-3-phosphate dehydrogenase; HRP: Horseradish peroxidase; ICAM: Intracellular adhesion molecule; IHC: Immunohistochemistry; IL-1a: Interleukin-1a; IL-6: Interleukin-6; IL-8: Interleukin-8; MCP-1: Monocyte chemotactic protein-1; PBS: Phosphate buffered saline; PHVPs: Polyurethane heart valve prostheses; PU: Polyurethane; RNA: Ribonucleic acid; RT: Room temperature; RT-PCR: Real time polymerase chain reaction; SC: Static cultivation; SMC-Myosin: Smooth muscle cell myosin; TEHVs: Tissue engineered heart valves; VCAM: Vascular cell adhesion molecule.

## Competing interests

The authors declare that they have no competing interests.

## Authors' contributions

GA mainly conducted experiments. TH wrote the manuscript, performed data analyses and statistical evaluations. NT also conducted experiments. UH supported the data generation. FK constructed the pulsatile bioreactor. CF manufactured the polymer scaffolds by spraying. MD was supervising the PHVP development and was one of the inventors of the 3-dimensional scaffolds. EW supervised the technical construction done by FK. BR was one of the inventors of the 3-dimensional scaffolds. CS supported this study from the medical point of view and supervised GA. BA conceived the experimental study, was the project owner; the device inventor, the group leader and overall supervisor. All authors read and approved the final manuscript.
